# Imbalances in Kynurenines as Potential Biomarkers in the Diagnosis and Treatment of Psychiatric Disorders

**DOI:** 10.3389/fpsyt.2022.913303

**Published:** 2022-06-28

**Authors:** Aye-Mu Myint, Angelos Halaris

**Affiliations:** Department of Psychiatry, Loyola University School of Medicine and Loyola University Medical Center, Maywood, IL, United States

**Keywords:** kynurenine, glutamate, GABA, treatment resistant depression (TRD), esketamine

## Introduction

Psychiatric disorders are heterogeneous in many aspects. Due to this heterogeneity, many hypotheses have been proposed especially in regards to understanding the etiopathology of these disorders. A variety of molecules and their associated systems have been proposed as presumptive etiological factors in different psychiatric disorders. Based on some of these hypotheses, many pharmacological agents have been developed over the course of over half a century. It is impossible to state whether a certain hypothesis is right or wrong. Each hypothesis may be applicable to certain individuals, for a certain condition and/or specific symptoms. In the majority of psychiatric syndromes, more than a single system is involved in the pathophysiology of the disorder in a given individual. As a matter of fact, in most of these patients, an array of different molecules act synergistically as a network, at times facilitatory and at times inhibitory. However, there are certain molecules or systems that play a key role in the network of several molecules. For example, chronic subclinical imbalances in the immune system is such a key etiopathogical determinant. Furthermore, imbalances in the kynurenine pathway resulting mainly through imbalances in the immune system, play a key role in dysregulations in several other systems, such as serotonergic, glutamatergic, dopaminergic, noradrenergic and Gamma amino-butyric acid (GABAergic) neurotransmissions (1).

**Figure 1:**
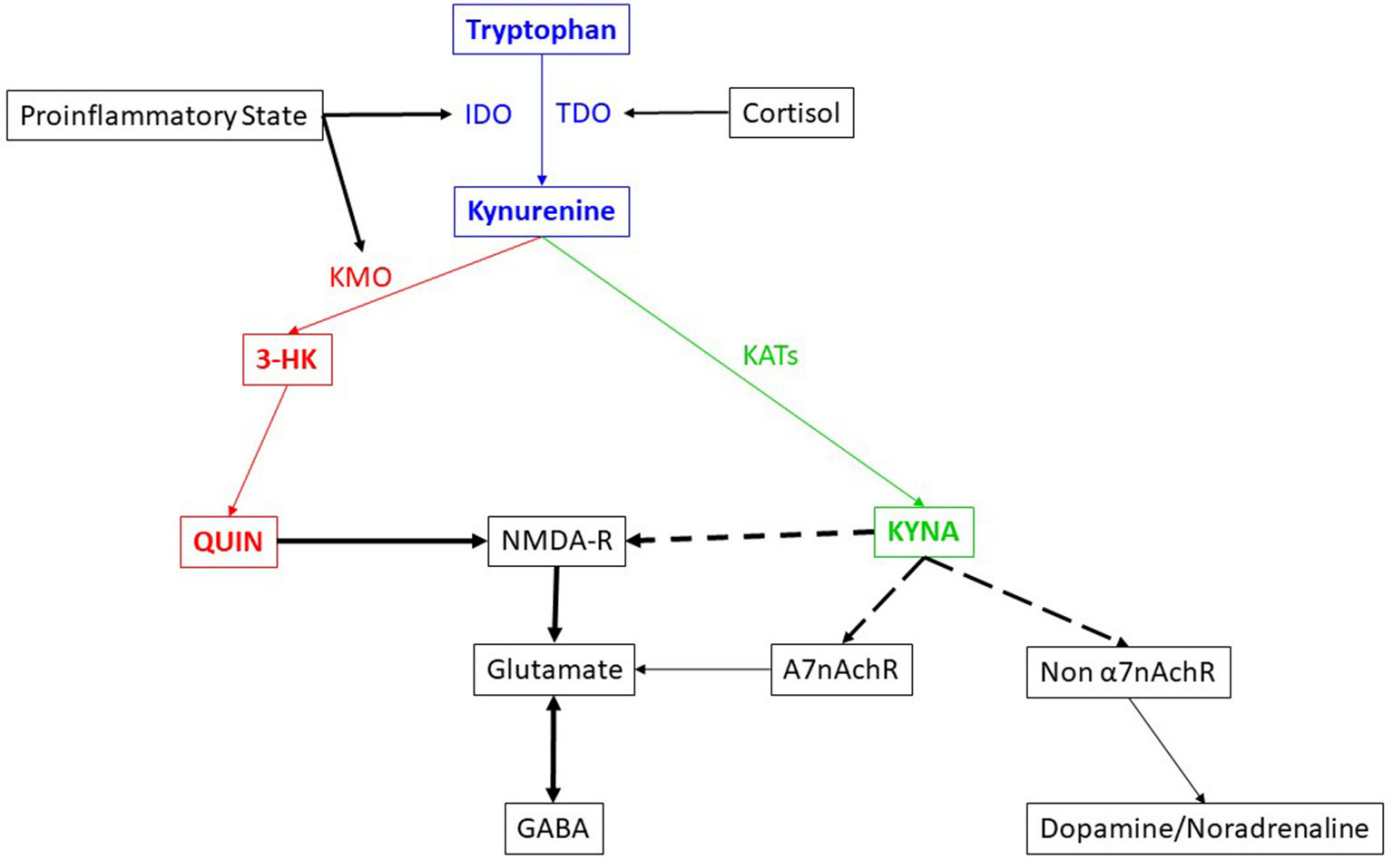
Brief description of the kynurenine pathway and its interaction with other systems.

The kynurenine pathway (Figure 1) has been reported to be involved in several psychiatric disorders (2–6). Medications, such as escitalopram, reportedly reduce neurotoxicity in depression (7). Recently, based on knowledge of the kynurenine pathway, and involvement of NMDA-R in chronic depression and suicide, a new medication which acts through NMDA-R antagonism, esketamine nasal spray, was developed and approved by the United States Food and Drug Administration (FDA) for treatment resistant depression (8) and in emergency situations to prevent suicide in severe depression (9). Based on the fact that the kynurenine pathway has a huge network with other neurochemical pathways, questions remain whether esketamine might also be therapeutically useful in other psychiatric conditions.

In this minireview, we analyzed the knowledge and information we have regarding the involvement of the kynurenine pathway in different psychiatric disorders, its complex interactions with other neuronal and neurochemical systems, and the introduction of innovative therapeutic agents, notably esketamine, and its possible therapeutic roles since esketamine is the only currently available medication which is directly link to the role of kynurenine pathway in psychiatric disorders. Nevertheless, the role of timely administration of anti-inflammatory agents is also discussed as a preventive measure for treatment resistant depression.

The blue, green and red colored text and arrows indicate the metabolic pathway itself. Blue color depicts the neutral nature in terms of neurotoxicity while the red color depicts neurotoxicity and green color depicts neuroprotection. The black color lines and texts indicate the network. The dotted arrows depict the inhibitory effect, while the complete arrows depict the excitatory or stimulatory effect. The double headed arrow indicate the antagonism in action. The thickness of the arrow indicates the strength of the effect.

## Kynurenine Pathway in Relation to Other Neurochemicals

### Kynurenine Pathway and Serotonergic System

The kynurenine pathway is directly related to the serotonergic system. Serotonin is synthesized from tryptophan which is an essential amino acid. This amino acid can also be degraded into kynurenine in the tryptophan metabolic pathway. This degradation can occur through one of two enzymes, tryptophan 2,3-dioxygenase (TDO) or indolamine 2,3-dioxygenase (IDO) (10–12). The enzyme TDO is activated by the stress hormone cortisol (13, 14), while the IDO enzyme is activated by the pro-inflammatory cytokines, such as interferon γ (IFN γ), interleukin 6 (IL6) and tumor necrosis factor α (TNF α) (15, 16). Therefore, chronic inflammation or the unregulated chronic imbalance in the immune system, as well as stress can activate the tryptophan degradation pathway, the kynurenine pathway. This increased degradation of tryptophan will lead to reduction in the availability of tryptophan for serotonin synthesis and ultimately reduction in the synthesis of serotonin. Serotonergic deficiency has been closely associated with depressive mood, and reversal of serotonergic deficiency by serotonin reuptake inhibitors (SSRI's) restores mood at least in a percentage of depressed patients.

Kynurenine (KYN), the degradation product of tryptophan by the enzymes, IDO or TDO, is further degraded to kynurenic acid (KYNA) via kynurenine amino transferases (KATs), or to 3-hydroxy kynurenine (3-HK) by kynurenine monooxygenase (KMO). 3-HK is further degraded to quinolinic acid (QUIN), the final product of which is nicotinamide adenine dinucleotide (NAD). QUIN is the antagonist of the NMDA type of the glutamate receptor. NMDA receptors are important in information processing in neurons, as well as in toxicity (17). On the other hand, KYNA is a NMDA-R agonist and considered to be neuroprotective against QUIN induced neurotoxicity (18). Since KMO enzymatic activity is enhanced by the pro-inflammatory cytokine, IFN γ, formation of QUIN is enhanced in cases of chronic immune activation. KMO activity is suppressed by the anti-inflammatory cytokine, IL4 (19). Enhanced QUIN can induce excitotoxicity through the NMDA-R and, in turn, neurotoxicity. These processes might, at least partially, contribute to treatment resistance or chronicity in depression and expand our understanding of the etiopathology of depression beyond serotonin depletion (20). In addition, this mechanism may not be confined only to depression since other disorders such as schizophrenia and bipolar disorders are also chronic disorders.

### Kynurenine Pathway and Glutamatergic System

Since QUIN is the antagonist of the NMDA type of the glutamate receptor, when there is chronic uncorrected immune activation, the formation of QUIN is enhanced and its synergistic agonistic action to Glutamate at the NMDA-R is also enhanced. This increase of QUIN can occur with or without increase in KYNA, the metabolite from the other arm of the KYN pathway. Sometimes, KYNA becomes even decreased, since the pathway is shifted to the arm of QUIN and in this manner, QUIN can induce neurotoxicity. Although this outcome might not be directly involved in inducing the symptoms of depression or schizophrenia or bipolar disorder or any specific symptoms of a psychiatric disorder, it could definitely induce neurotoxicity and neurodegeneration which could eventually result in treatment resistance or chronicity of the psychiatric illness (20).

However, KYNA which is a NMDA-R antagonist, and is generally considered as a protective metabolite against QUIN (21), can also be toxic, since it is also an antagonist of all ionotropic excitatory amino acid receptor activities (19). Its abnormal accumulation beyond physiological levels could induce glutamatergic hypo-functioning and might diminish cognitive function (22). Moreover, while one of the tryptophan metabolites, 5-hydroxyindole (5HI), activates the α7-nicotinic acetylcholine receptor (α7nAchR) and induces glutamate release (23, 24), KYNA is an antagonist of a7nAchR (25). Since KYNA downregulates the permissive role of 5HI activation at the α 7nAchR, the accumulation of KYNA could suppress α7nAchR function and induce disruption of auditory sensory gating (26). This is considered to be a causal factor in psychotic symptoms development.

In summary, the above referenced imbalances in the metabolism of the kynurenine pathway play an important role in psychiatric disorders in regards to aspects of acute psychotic symptom development as well as chronicity or treatment resistance. In addition, the neurotoxicity and cognitive disturbances through the enhanced degradation of KYN into KYNA and QUIN might play a role in neurodegenerative disorders. This would explain the connection between depression and dementia.

### Kynurenine Pathway and Dopaminergic/Noradrenergic System

In addition, 5HI inhibits the non- α7nAchR mediated release of noradrenaline, dopamine and acetylcholine (27), while KYNA regulates the activity and expression of non-a7nAchR based on dosage and length of exposure (25). Through this action, the abnormal accumulation of KYNA might disturb dopaminergic and noradrenergic neurotransmission as well. It has long been known that noradrenergic and dopaminergic neurotransmission play important roles in both mood disorders and schizophrenia.

### Kynurenine Pathway and GABAergic System

Although not through the direct action of kynurenines on the GABAergic pathway, the kynurenine pathway has an association with GABAergic neurotransmission through its NMDA receptor activity. In this subsection, the interaction between GABAergic and glutamatergic transmission in general, and its associations with psychiatric and neuropsychiatric disorders will be briefly described.

To maintain optimal central nervous system (CNS) function, a balance between excitatory and inhibitory synaptic transmission is essential for long-term stability and function of neuronal networks. Traditionally, excitatory and inhibitory neurotransmission systems have been associated with the glutamatergic and GABAergic systems, respectively. Homeostatic synaptic plasticity depends on signaling cascades regulating in parallel the efficacy of glutamatergic and GABAergic transmission and homeostatic synaptic plasticity (28). In this context, glutamine (Gln) is a precursor of several neurotransmitter amino acids, notably. the excitatory amino acids, glutamate (Glu) and aspartate (Asp), and the inhibitory amino acid, γ-amino butyric acid (GABA). Gln is present in the CNS and participates in a variety of metabolic pathways. Disturbances of Gln metabolism and/or transport contribute to changes in glutamatergic or GABAergic transmission associated with brain pathology (29).

Dysfunction of the GABAergic/glutamatergic network in telencephalic brain structures may be the major pathogenetic mechanism underlying psychotic symptoms in schizophrenia and bipolar disorder. GABAergic neuropathology may underlie the disturbance of the reciprocal interaction between GABAergic interneurons and principal glutamatergic pyramidal neurons and this may induce positive and negative symptoms and cognitive dysfunction that are observed in psychotic patients (30, 31). Many studies have suggested that schizophrenia and bipolar disorder are diseases characterized by a deficit of GABAergic transmission with consequent glutamatergic and monoaminergic network dysfunction (32–34).

Stress has long been recognized as a major contributory factor to medical and psychiatric illnesses. Prolonged stress and different forms of experimental stress suppress the inhibitory action of GABA, thereby causing hyperactivation of glutamatergic stimulation of Corticotrophin releasing factor (CRF) producing cells and exhaustion of the hypothalamo-pituitary-adrenal (HPA) axis resulting in low levels of glucocorticoids. This is sometimes referred to as “the GABAergic deficit hypothesis of major depressive disorder” (35, 36). Since cortisol, a stress hormone enhances the tryptophan degradation, increased formation of kynurenine pathway metabolites plays a role in enhanced glutamatergic neurotransmission through NMDA-R activity.

An imbalance between excitatory and inhibitory neurotransmission systems may also underlie the synaptic dysfunction caused by β-amyloid (Aβ) peptides (37–40). Pharmacological treatments aimed at modulating excitatory and/or inhibitory neurotransmission may be helpful in improving symptoms of Alzheimer's Disease (AD). It has been suggested that strategies aimed at reestablishing the balance between both systems, particularly in early stages of AD, may be effective in halting, if not reversing, the functional deficits caused by Aβ (40–42). The correlation between kynurenine pathway (KP) metabolites and AD with major emphasis on its two functionally contrasting neuroactive metabolites, KYNA and QUIN the reader is referred to this review article (43).

A significant non-invasive imaging tool that is presently available to elucidate brain biochemistry, especially as it relates to the highly complex neuronal pathways, such the pathways summarily reviewed in this article, is magnetic resonance spectroscopy (MRS). Using ^13^C label, ^13^C MRS can visualize the role of glutamate/GABA neurotransmission in disorders referred to above, notably AD, schizophrenia, and bipolar disorder (44).

## Kynurenine Pathway Involvement in Psychiatric Disorders

### Mood Disorder

As stated earlier, in the case of a pro-inflammatory state and IDO activation, there will be reduced serotonin availability for serotonergic neurotransmission. The pro-inflammatory status in major depressive disorder, documented in numerous studies, would activate not only IDO but also KMO enzyme activities and this activation may in turn shift KYN metabolism to the 3-HK and QUIN arm. It has been proposed that the increase in these toxic metabolites, may make the astrocyte–microglia–neuronal network vulnerable to environmental factors, such as stress. It has also been proposed that an impaired glial-neuronal network, induced by the unbalanced KYN pathway, may contribute to the recurrence and chronicity of major depressive disorder (20). The neurotoxic metabolites may then induce astrocytic and neuronal apoptosis, which would weaken the function of the glial–neuronal network. Loss of astrocyte function could also impair glutamate–glutamine metabolism through the glutamine synthetase enzyme, which occurs mainly in astrocytes (45).

In medication-naïve or medication-free patients with major depressive disorder, an imbalance has been demonstrated between these neuroprotective and neurotoxic pathways, with reduction in the protective metabolite, KYNA (2). The ratio between KYNA and KYN, which indicates how much of the KYN would be degraded into KYNA, was significantly lower in depressed patients than healthy controls. After a 6-week medication trail with currently available antidepressants, mainly selective serotonin reuptake inhibitors (SSRI's), the metabolic imbalance in the KYN pathway could not be reversed. This imbalance might, in the long term, induce neurodegenerative changes and these, in turn, might induce chronicity and treatment resistance. In our study (5) on QUIN immunoreactivity in post-mortem brain tissues from patients with major depressive disorder or bipolar depression and normal controls, we demonstrated that QUIN immunoreactivity was increased in the prefrontal cortex area in the brains of patients with major depression and patients with bipolar depression who committed suicide. However, in our postmortem immunohistochemical investigation, we observed decreased QUIN immuno-stained microglia in the hippocampal area of the depressed patients (46). This could be due to the fact that QUIN enhanced expression in the brain is area specific, or, that there was a failure to detect QUIN expression due to cell loss in hippocampal area. Another study on KYN metabolites concentrations in the cerebrospinal fluid (CSF) of suicidal patients with different psychiatric disorders also demonstrated the increase in QUIN concentration regardless of the psychiatric disorders (47). This indicates that the KYN metabolites imbalance in the brain in suicidal patients is not limited only to major depression.

### Kynurenine Pathway Involvement in Schizophrenia

Regarding the KYN pathway in schizophrenia, a study of KYNA concentrations in post-mortem brain tissue in different cortical regions revealed increased KYNA in the samples from schizophrenic patients compared with a control sample, particularly in the prefrontal cortex (48). In another investigation of the anterior cingulate cortex, a small and non-significant increase of KYNA in medicated schizophrenics was observed (49). Our postmortem histochemical study in brain tissue from chronically medicated patients showed reduced QUIN staining in the hippocampal area (50). These studies raised the question of whether the increase in KYNA might be associated with antipsychotic medication. However, increased levels of KYNA were also observed in the cerebrospinal fluid of schizophrenic patients (51). Since most of the patients in this study were drug-naïve first-episode patients, this increase could not have been caused by antipsychotic treatment. It was hypothesized that accumulation of KYNA may lead to schizophrenic symptoms (52). Our finding in medication-naïve schizophrenic patients indicated increased plasma 3-HK and decreased plasma KYNA compared to healthy controls, and this was reversed after 6 weeks of antipsychotic treatment. This finding would be an indirect indicator of the accumulation of 3-HK due to enhanced KMO activity (4) in those patients which might further lead to increased QUIN formation. In addition, a recent study on KYN metabolites in brain tissue failed to show either increased KYNA or decreased QUIN in schizophrenia (53). Thus, the findings regarding KYN metabolites in schizophrenia are inconclusive.

While increased KYNA could induce psychotic symptoms, increased QUIN could also induce neurotoxicity and probably also suicide. Therefore, it is not very conclusive in management of schizophrenia via manipulation of KYN metabolites.

### Kynurenine Pathway and Drug Discoveries

Based on all findings from basic, preclinical and clinical studies, ketamine, an NMDA antagonistic medication, has received major interest over the last two decades (54, 55). A study has demonstrated that a single infusion of ketamine could induce reduction in Montgomery Asberg Depression Rating Score (MADRS) and suicidal ideation score in treatment resistant patients with major depression or bipolar depression (56). An even stronger but short-term effect was demonstrated with (S)-ketamine, an enantiomer of ketamine (9). Although (S)-ketamine is four times more potent at the NMDA receptor than another enantiomer (R)-ketamine (57), the (R)-isoform showed a longer-term effect on depressive symptom reduction (58). The improvement of suicidal ideation was also documented with intranasal ketamine (59). In 2019, esketamine as an intranasal antidepressant for treatment resistant depression, was approved by FDA (8). Esketamine is also now approved for use in major depressive disorder patients presenting with suicidal ideation or intent.

As discussed above, increased QUIN concentration in CSF of suicidal patients is not limited to major depression. A question could be raised whether indications for the use of esketamine could be expanded to other psychiatric emergency conditions beyond major depression.

## Future Perspectives

In previous studies, both QUIN immunohistochemical expression in the anterior cingulate cortex (5) and increased QUIN CSF concentration (47) were documented in suicidal patients with different psychiatric disorders. However, in hippocampus of both depressed and schizophrenic patients in general (both suicide victims and those who died due to other causes), we could not demonstrate increased QUIN immunohistochemical expression (46, 50), but we found decreased expression instead. This raises the question whether the imbalance in QUIN expression between different brain areas could induce compulsive and aggressive behavior, such as suicide in those patients with psychiatric disorders, notably mood and schizophrenic disorders. If this were to be the case, further opportunities could present themselves in drug development. Carefully and closely observed clinical studies should be performed to achieve the preventive treatment for suicide in both depressive disorders and psychotic disorders.

Moreover, based on the findings which indicated the role of inflammatory state in inducing increased tryptophan degradation and imbalance in KYN metabolites, to prevent the development of treatment resistance in psychiatric disorders through timely use of anti-inflammatory medication should also be considered.

Since it is not easy to analyze the KYN metabolites in the specific brain regions, new invention in imaging technology to achieve a surrogate marker for this imbalance in the KYN metabolites in the different brain region could be very useful to take preventive measures for suicide and neurotoxicity.

## Author Contributions

Both authors contributed to the concept, design, and writing of this mini review article. Both authors contributed to the article and approved the submitted version.

## Conflict of Interest

The authors declare that the research was conducted in the absence of any commercial or financial relationships that could be construed as a potential conflict of interest.

## Publisher's Note

All claims expressed in this article are solely those of the authors and do not necessarily represent those of their affiliated organizations, or those of the publisher, the editors and the reviewers. Any product that may be evaluated in this article, or claim that may be made by its manufacturer, is not guaranteed or endorsed by the publisher.
